# The Eating Disorders Genetics Initiative 2 (EDGI2): study protocol

**DOI:** 10.1186/s12888-025-06777-5

**Published:** 2025-05-26

**Authors:** Natasha Berthold, Casey M. MacDermod, Laura M. Thornton, Richard Parker, Shantal Anid Cortés Morales, Liv Hog, Hannah L. Kennedy, Jerry Guintivano, Patrick F. Sullivan, James J. Crowley, Jessica S. Johnson, Andreas Birgegård, Bengt T. Fundín, Emma Frans, Jiayi Xu, Michaela Pettie “Ngāti Pūkenga”, Allison L. Miller, Mariana Valdez Aguilar, Sarah Barakat, Mohamed Abdulkadir, Jennifer P. White, Janne T. Larsen, Elsie Trujillo, Bertha Winterman, Ruyue Zhang, Rachel Lawson, Stephen Wonderlich, Joseph Wonderlich, Lauren M. Schaefer, Philip S. Mehler, Judy Oakes, Marina Foster, Jennifer Gaudiani, Eva Trujillo Chi Vacuán, Emilio J. Compte, Liselotte V. Petersen, Zeynep Yilmaz, Nadia Micali, Jennifer Jordan, Martin A. Kennedy, Sarah Maguire, Laura M. Huckins, Yi Lu, Lisa Dinkler, Nicholas G. Martin, Cynthia M. Bulik

**Affiliations:** 1 Department of Psychiatry, University of North Carolina at Chapel Hill, #7160, 101 Manning Drive, Chapel Hill, CBNC 27599 - 7160 USA; 2 School of Human Sciences, University of Western Australia, Crawley, WA 6009 Australia; 3 Perron Research Institute, Nedlands, WA 6009 Australia; 4 QIMR Berghofer Medical Research Institute, Royal Brisbane Hospital, Locked Bag 2000, Brisbane, QLD 4029 Australia; 5 Research Department, Comenzar de Nuevo, Monterrey, Mexico; 6 Tecnologico de Monterrey, Escuela de Medicina y Ciencias de la Salud – , Monterrey, Mexico; 7 Department of Medical Epidemiology and Biostatistics, Karolinska Institutet, 171 77 Stockholm, Sweden; 8 Department of Pathology and Biomedical Science, University of Otago, Christchurch, New Zealand; 9 Department of Genetics, University of North Carolina at Chapel Hill, Chapel Hill, NC 27599 USA; 10 Division of Molecular Psychiatry, Department of Psychiatry, Yale University, New Haven, CT USA; 11 Insideout Institute for Eating Disorders, The University of Sydney, Sydney, Australia; 12 Department of Public Health, National Centre for Register-Based Research, Aarhus University, Aarhus, Denmark; 13 Department of Psychology, University of Albany, State University of New York, Albany, NY USA; 14 South Island Eating Disorders Service, Health NZ Te Whatu Ora, Christchurch, New Zealand; 15 Center for Biobehavioral Research, Sanford Health, Fargo, ND USA; 16 Department of Psychiatry and Behavioral Science, University of North Dakota School of Medicine and Health Sciences, Fargo, ND USA; 17 ACUTE Center for Eating Disorders and Severe Malnutrition, Denver Health and Hospital Authority, Denver, CO USA; 18 Gaudiani Clinic, Denver, CO USA; 19 Eating Behavior Research Center, School of Psychology, Universidad Adolfo Ibáñez, Santiago, Chile; 20 Department of Biomedicine, Aarhus University, Aarhus, Denmark; 21 Center for Eating and Feeding Disorders Research, Mental Health Services of the Capital Region of Denmark, Psychiatric Centre Ballerup, Copenhagen, Denmark; 22 Great Ormond Street Institute of Child Health, University College London, London, UK; 23 Department of Psychological Medicine, University of Otago, Christchurch, New Zealand; 24 Health NZ - Te Whatu Ora, Christchurch, New Zealand; 25 Faculty of Medicine and Health, University of Sydney, Sydney, Australia; 26 Department of Nutrition, University of North Carolina at Chapel Hill, Chapel Hill, NC 27599 USA

**Keywords:** Anorexia nervosa, Bulimia nervosa, Binge-eating disorder, Avoidant/restrictive food intake disorder, Genome-wide association, Psychiatric genetics

## Abstract

**Background:**

The Eating Disorders Genetics Initiative 2 (EDGI2) is designed to explore the role of genes and environment in anorexia nervosa, bulimia nervosa, binge-eating disorder, and avoidant/restrictive food intake disorder (ARFID) with a focus on broad population representation and severe and/or longstanding illness.

**Methods:**

A total of 20,000 new participants (18,700 cases and 1,300 controls) will be ascertained from the United States (US), Mexico (MX), Australia (AU), Aotearoa New Zealand (NZ), Sweden (SE), and Denmark (DK). Comprehensive phenotyping and genotyping will be performed for participants in US, MX, AU, NZ, and SE using the EDGI2 questionnaire battery and participant saliva samples. In DK, case identification and genotyping will be through the National Patient Register and bloodspots archived near birth. Case–control and case-case genome-wide association studies will be conducted within EDGI2 and enhanced via meta-analysis with external data from the Eating Disorders Working Group of the Psychiatric Genomics Consortium (PGC-ED). Additional analyses will explore genetic correlations between eating disorders (EDs) and other psychiatric and metabolic traits, calculate polygenic risk scores (PRS), and leverage functional biology to evaluate clinical outcomes. Moreover, analyzing PRS for patient stratification and linking identified risk loci to clinically relevant phenotypes highlight the potential of EDGI2 for clinical translation.

**Discussion:**

EDGI2 is a global expansion of the EDGI study to increase sample size, increase participant representation across multiple ancestral background*s*, and to include ARFID. ED genetics research has historically lagged behind other psychiatric disorders, and EDGI2 is designed to rapidly advance the study of the genetics of the major EDs. Exploring EDs at both the diagnostic level and the symptom level will provide an unprecedented look at the genetic architecture underlying EDs.

**Trial registration:**

EDGI2 is a registered clinical trial: clinicaltrials.gov NCT06594913. https://clinicaltrials.gov/study/NCT06594913 (posted September 19, 2024).

## Background

We describe the *Eating Disorders Genetics Initiative 2* (*EDGI2*), a multi-national study designed to expand genomic discovery across four major eating disorders (EDs) (including AN [anorexia nervosa], BN [bulimia nervosa], BED [binge-eating disorder], and ARFID [avoidant/restrictive food intake disorder]). EDGI2 expands the work of EDGI [1] and the Eating Disorders Working Group of the Psychiatric Genomics Consortium (PGC-ED), and previous investigations including the Anorexia Nervosa Genetics Initiative (ANGI) [2] that identified eight significant loci associated with AN and intriguing genetic correlations (*r*_*g*_) with both psychiatric and metabolic/anthropometric traits, strongly suggesting that AN is a metabo-psychiatric disorder [3]. A genome-wide association study (GWAS) on binge-eating behavior suggested similar psychiatric genetic correlations as AN but divergent direction of metabolic/anthropometric genetic correlations [4]. EDGI2 expansion emphasizes recruitment of a broader range of people with EDs and a particular focus on people with longstanding and/or severe forms of the illnesses whose genomes may be enriched for causal variants.

## Methods

### Study aims

#### Aim 1: Extend EDGI core business by increasing sample size, populations, and ED phenotypes

We will ascertain, phenotype, and collect biosamples from ~ 20,000 people with AN, BN, BED, and/or ARFID (i.e., cases) and controls, increasing the total meta-analysis sample size to ~ 80,000 cases and ~ 730,000 controls. Collaboration with relevant groups will help attain the goal of enrolling 30% of cases from groups that have been less represented in previous ED research. EDGI2 also focuses on enrolling people with severe and/or longstanding AN, as their DNA may reflect higher genetic burden.

#### Aim 2: Apply statistical genetic analyses to better understand the heterogeneity and underlying biology of EDs

A multistage approach will implement a state-of-the-science pipeline for ED genomics. Primary analyses will include a GWAS of diagnoses, component behaviors, and dimensional traits, followed by case-case comparison, SNP-heritability (SNP-h^2^), genetic correlations (*r*_*g*_), polygenic risk scores (PRS), rare copy number variant (CNV) analyses, and bioinformatic integration for meaningful translation of GWAS results. Detailed phenotypes and PRS will be used for patient stratification, comparing standard diagnostic approaches with a novel, data-driven taxonomy of EDs. Finally, we will evaluate the hypothesis that AN is a "metabo-psychiatric disorder" by clarifying its relationship with metabolic and anthropometric traits through linkage disequilibrium score regression (LDSC), pathway-based PRS analysis (PRSet), phenome wide association study (PheWAS), Mendelian randomization (MR), and other emerging methods.

#### Aim 3: Evaluate genetic and environmental risk and resilience factors to inform risk prediction

Genetic or molecular groupings and patterns across cases and controls will be investigated through phenotypically characterizing individuals in the highest and lowest deciles of PRS based on clinical and phenotypic outcomes, and by genetically characterizing the top and bottom deciles of clinical severity across various PRS and PRS pathways. We will compare high-PRS cases with high-PRS controls to identify potentially protective environmental and genetic factors.

#### Aim 4: Determine where in the body EDs “live.”

First, the "Gwas2cells" method will be used to identify brain cell types and anatomical regions implicated by genomic studies of EDs, and these findings will be contrasted with those for psychiatric disorders, neurological diseases, metabolic diseases, and brain-related traits. We will then identify gene-tissue associations across disease-relevant tissues and cell types, and leverage single-nucleus RNA sequencing (snRNA-seq) atlases to pinpoint brain cell types strongly implicated in the genomics of each ED and predict genetically regulated gene expression (GREx) in ED-relevant tissues and cells. Dynamic GREx will model gene expression in ED-relevant contexts, such as sex, body mass index (BMI),^1^ or stress, enabling precise and personalized modeling of gene expression changes under hypothetical scenarios like weight gain/loss, stressful life events, or depression.

#### Aim 5: Translational Summit

A Translational Summit in the final year will include leaders from genomics, neuroscience, interventional psychiatry, pharma, treatment, and community stakeholders. The summit will focus on strategies for optimizing and channeling the results of the study into a translational roadmap aimed at enhancing the prevention and treatment of EDs.

### Participants

EDGI2 will recruit 20,000 participants from the United States (US), Mexico (MX), Australia (AU), New Zealand (NZ), Sweden (SE), and Denmark (DK); within this recruitment target we will enroll ~ 3000 individuals with longstanding and/or severe AN. Effort will be made to enroll individuals who have been less frequently studied including those with varying body sizes and males. The minimum adult age for inclusion will vary by country: 16 in NZ and SE; 18 in US, AU, and MX; and 21 in Puerto Rico, with no upper age limit. Child case participants will be included for MX (15–17 years of age) and must have parent/guardian consent to participate. DK participants will all be born in DK between 1981 and 2008.

### Inclusion criteria

#### Case definitions

Participants from US, MX, AU, NZ, and SE will be selected based on self-reported lifetime Diagnostic and Statistical Manual of Mental Disorders-fifth edition (DSM- 5) criteria for AN, BN, BED, and/or ARFID via the ED100K.v4 ED online questionnaire, revised from the previous version (ED100K.v3) to include ARFID [2]. The ARFID portion contains items developed by the study team in addition to adapted, lifetime versions of the Nine Item ARFID Screen (NIAS) [5] and Pica, ARFID, and Rumination Disorder ARFID Questionnaire (PARDI-AR-Q) [6]. In MX, those recruited through the Comenzar de Nuevo clinic will be identified based on clinical diagnosis. In DK, cases of AN and BN will be identified using International Classification of Diseases – 10 th Revision (ICD-10) codes (F50.0, F50.1 for AN; F50.2, F50.3 for BN). DK will not include ARFID or BED as no ICD-10 codes exist for these disorders. SE will link participants to diagnostic records from the Swedish National Patient Register and Quality Register (Riksät) that have been validated through diagnostic interviews with acceptable reliability [7].

For US, MX, AU, NZ, and SE, longstanding and/or severe AN (restricting or binge/purge AN subtype) will be captured by the EDGI2 battery and include AN duration ≥ 5 years, lowest illness-related BMI ≤ 15, ≥ 3 treatment periods, and above age 20 at enrollment. Severe cases with rapid weight loss who attain very low body weights will also be included. DK will use the Anorexia Nervosa Register-based Severity Index (AN-RSI), which creates a systematic and weighted combination of age at onset, inpatient treatments, outpatient readmissions, treatment length, and illness duration [8]. Modifications to the AN-RSI definition may be made based on emerging research.

#### Control definitions

In the US, MX, and SE, controls will be selected based on the absence of lifetime ED symptoms as determined via the ED100K.v4 [2]. Controls must have a minimum lifetime adult BMI ≥ 18.55 and maximum lifetime BMI < 30 and no history of binge eating, compensatory behaviors, use of glucagon-like peptide-1 (GLP-1) receptor agonists, or atypical AN. They also cannot report a history of an ED in any first-degree relatives. For AU and NZ, controls will be selected from healthy donor samples in previously collected cohorts. In DK, control inclusion criteria will be the absence of lifetime AN, BN, or other ED diagnosis based on ICD-10 codes.

#### Recruitment

EDGI2 recruitment in US, MX, AU, NZ, and SE will be multi-faceted, targeting broad populations. Direct case recruitment will be conducted from clinics and treatment programs actively involved in the study in the US and MX. For MX, children/adolescents (15–17 years of age) will only be recruited from the study clinic (Comenzar de Nuevo). NZ and SE will not perform direct recruitment from treatment services; however, clinician networks in both public and private treatment services will be invited to advertise the study. Coordinated recruitment campaigns will be launched in traditional media, including press releases, television, and radio to raise awareness about EDGI2. An engaging social media presence will be further developed on Facebook, Instagram, Bluesky, LinkedIn, Reddit, Snapchat, YouTube, podcasts, and emerging platforms to reach people on platforms and applications where they are active online. To personalize and amplify recruitment efforts, US, AU, NZ, MX, and SE will engage in consultation with people familiar with EDs and other interested parties (e.g., friends, family, caregivers, support providers) to encourage participation as cases or controls. Outreach will prioritize spotlighting varying body sizes and populations affected by EDs.

Controls for US, MX, and SE will also be recruited through the traditional and social media campaigns, and through community events and universities. AU and NZ will not recruit new controls. DK cases and controls will be acquired from the Danish National Health Registry of all individuals born in DK between 1981–2008; new cases (not captured in EDGI [1] or ANGI [2]) will be individuals with first BN and/or AN register diagnoses between 2016-present. Each country conducting active recruitment will have a unique local EDGI2 website providing localized information, educational material, and resources.

### Procedure

#### Consent and pre-screen questionnaires

Slight procedural differences exist across countries. In general, potential participants are directed through a link on their site-specific websites or a QR-code to a prescreen capturing basic eligibility information including age and other items reflecting inclusion criteria. If they meet criteria on the prescreen, they are shown a country-specific consent. Consent and prescreen questionnaires are applied through REDCap in US, MX, AU, and NZ. In SE, consent is collected through digital identification linked to Swedish national identity numbers. In MX, parent/guardian consent and child assent are required for child/adolescent (ages 15–17) participation and are collected in person at the main study clinic and registered on REDCap.

NZ has tailored consent and data management procedures for participants identifying as Māori (Indigenous People of NZ) and/or Pacific Islands Peoples (Pasifika) to align with national guidelines for health research. This consent and data management process recognizes the interconnectedness of Māori and Pasifika participants across individuals and across time, while enabling participation in this important international research. Their phenotypic and genetic data will be shared internationally under controlled access after approval by a NZ specific data access committee. Additional detailed information is available by contacting the NZ principal investigators.

#### Self-report measures

Slight procedural differences exist across participating sites. Individuals who complete the prescreen and consent are then presented with the EDGI2 questionnaire battery. We describe the specific metrics below in the order in which they will be presented (Table 1). 

*Screening questionnaire.* Participants are first presented with the ED100K.v4 questionnaire to determine eligibility and case designation [2]. Participants who meet eligibility criteria are asked for contact information and sent a unique link via email for the core and additional questionnaires. Participants can save and exit questionnaires at any time and navigate back to the survey as desired. In SE, contact information will be given directly after consent.

**Table 1:** EDGI2 Assessment Battery (US, MX, AU, NZ, SE)

Questionnaire Section		Assessments Included	Domains Captured	Completed by
**Screening Questionnaire**		ED100K.v4 [2]. (including Lifetime Version of the Nine Item ARFID Screener [L-NIAS] [5], and Lifetime Version of the Pica, ARFID, and Rumination Disorder Interview – ARFID – Questionnaire [L-PARDI-AR-Q] [6] (adapted for this study)	Lifetime history of AN, BN, BED, and ARFID	All
**Core Questionnaires**		PACE Questionnaire (Polygenic Risk of Anorexia Nervosa and its Clinical Expression) [9]	Physical and emotional responses to feelings of hunger and fullness	All
		7-Item Binge-Eating Disorder Screener (BEDS- 7) [10]	Current BED symptoms	Case only
		Eating Disorder Examination-Questionnaire (EDE-Qv6) [11]	Current ED symptoms	Case only
		NIAS [5]	Current ARFID symptoms	All
		Muscle Dysmorphic Disorder Inventory (MDDI) [12]	Current symptoms of muscle dysmorphia	All
		Drive for Muscularity Scale (DMS) [13]	Current drive for muscularity	All
		Food Security (adapted for this study)[14]	Current symptoms of food insecurity	All
**Additional Questionnaires**	**Health and Treatment History**	Health History (developed for this study)	History of mental health, neurodevelopmental, gastrointestinal, and other disorders	All
		Item assessing ED-related nicotine use (developed for this study)	Lifetime nicotine use for the purposes of weight and shape modification	All
		ED-related stimulant use (developed for this study)	Lifetime stimulant use for the purposes of weight and shape modification	All
		ED Treatment History (developed for this study)	ED treatment history including barriers to care, tailored to site/country	Case only
		Menstruation (developed for this study)	Menstrual history	Female or intersex participants only
	**Mental Health and Behavior**	Obsessive Compulsive Inventory-Revised (OCI-R) [15]	Current obsessive–compulsive disorder symptoms	All
		Autism Spectrum Quotient- 10 (AQ- 10) [16]	Current symptoms associated with autism spectrum disorder	All
		Adult ADHD Self-Report Scale (ASRS) [17]	Current symptoms associated with ADHD	All
		Generalized Anxiety Disorder Screener (GAD- 7) [18]	Current anxiety symptoms	All
		Patient Health Questionnaire- 9 (PHQ- 9) [19]	Current depression symptoms	All
	**Lifetime Mood**	Depression, Mania, and Anxiety Sections of the Genetic Links to Anxiety and Depression (GLAD) Battery [20]	Lifetime symptoms of depression, mania, and anxiety	All
	**Self-Violence**^a^	Adapted Suicidal Ideation and Suicide Plan Sections of the Self-Injurious Thoughts and Behaviors Interview (SITBI) [21]	Lifetime suicidal ideation and suicide planning	All
		Adapted Suicidal Behavior Section of the Diagnostic Interview for Genetic Studies version 3.0 (DIGS) 3.0 [22]	Lifetime suicidal behavior	All
		Single-Item Non-Suicidal Self-Injury (NSSI) Screen (adapted from [23])	History of NSSI (yes or no)	All
		Adapted Inventory of Statements About Self-Injury (ISAS) [24]	Lifetime motivations for engaging in NSSI	All
	**Substance Use**	Adapted Lifetime Version of Alcohol Use Disorders Identification Test (AUDIT) [25]	Lifetime alcohol use	All
		Heaviness of Smoking Questionnaire [26]	Lifetime assessment of smoking	All
		Heaviness of Vaping Questionnaire [26]	Lifetime assessment of vaping	All
		Adapted Lifetime Version of Drug Use Disorders Identification Test (DUDIT) [27]	Lifetime drug use	All
	**Life Events**	Life Events Checklist for DSM- 5 (LEC- 5) (Adapted from [28])	Life events and trauma history, and emotional and physical neglect/abuse during childhood	All
	**ED-QOL**	ED Quality of Life (ED-QOL) [29, 30]	ED-specific health-related quality of life	Case only
	**CET**	Compulsive Exercise Test (CET) [31]	Compulsive and driven exercise	All
	**Perfectionism**	Multidimensional Perfectionism Scale (MPS) [32]	Perfectionism	All
	**CDE for Adults**	DSM- 5 Level 1 Cross-Cutting Questionnaire [33]	Current mental health domains that are important across psychiatric diagnoses	All
		World Health Organisation Disability Assessment Schedule version 2.0 (WHODAS v2.0) [34]	Current difficulties due to health conditions	All adult participants
	**CDE for Youth**^b^	DSM- 5 Level 1 Cross-Cutting for Youth version 1[35]	Current mental health domains that are important across psychiatric diagnoses, altered for youth assessment	All child participants
		Revised Children’s Anxiety and Depression Scale- 25 version 1.0 (RCADS- 25 v1.0)[36]	Measurement of current anxiety and low mood in youth	All child participants

EDGI2 questionnaire battery ordered by questionnaire section. ‘Domains captured’ describes the phenotype level information elicited by the questionnaire, and ‘completed by’ identifies which participants fill out the questionnaire, all (cases and controls), cases only, or participants in specific demographic groups

^a^excluded in some countries

^b^MX only

*Core eating disorder questionnaires*. The core battery includes validated questionnaires to measure eating behaviors and ED symptomology (Table 1). The questionnaires comprehensively phenotype AN, BN, BED, and ARFID, as well as transdiagnostic ED symptoms such as weight and shape concern and binge-eating behavior. Questions capturing muscle dysmorphia and food insecurity have been added based on community feedback. We hope to include atypical AN in the future. Participants in US and NZ receive gift card compensation upon completing all screening and core questionnaires and returning their saliva sample; in MX and SE, participants complete all questionnaires and provide a sample prior to receiving incentives; and incentives are not provided in AU.

*Additional Questionnaires.* Additional questionnaires capture 10 domains across physical health, mental health, substance use, treatment experiences and access (adapted for each country), exercise, and quality of life (Table 1). These additional questionnaires capture critically relevant information that informs a deeper phenotypic analysis, while also maintaining a survey length that does not overburden the participant.

#### Site specific questionnaires

*Sweden*. The EDGI2 battery has been linguistically translated into Swedish and culturally adapted for SE. SE-specific questionnaires address the impact of ED on social relationships, work, income, and education; participants’ perceived reasons for ED development; and factors supporting recovery. An additional Substance Use Questionnaire captures “snus” use (Swedish oral nicotine pouches). In addition to ED-QOL, quality of life is captured by the EuroQol-5 Dimension 5-level version questionnaire (EQ- 5D- 5L) [37]. Individuals who score > 3 on the AQ questionnaire also complete the Comprehensive Autistic Trait Inventory [38].

*Mexico*. The EDGI2 battery has been linguistically translated into Spanish and culturally adapted for MX. *Screening questionnaire*. This is only used to determine eligibility in adults who participate outside of Comenzar de Nuevo. All participants complete the Core Questionnaires. Adult participants complete all additional questionnaires, including CDE for adults. Participants 15–17 years complete all additional questionnaires except Self-Violence and the DUDIT portion of the Substance Use questionnaire. They will also complete CDE for youth.

*Australia and New Zealand*. AU and NZ are administering all questionnaires in the EDGI2 battery excluding the Self-Violence section of the additional questionnaires. NZ and AU have made country-specific and cultural adjustments to the Health and Treatment section.

#### Bio-sampling

Following completion of the ED100K.v4 and provision of contact information, eligible participants are sent a saliva sample collection kit via courier or mail (US, AU, NZ, SE). This includes a welcome letter, the saliva sample collection kit, site-specific instructions, and a pre-paid return envelope addressed to the participant’s respective collection site. For in-patient participants enrolled through some independent centers (e.g., ACUTE Center for Eating Disorders and Severe Malnutrition, Denver, CO and Sanford Health, Fargo, ND) the saliva sample collection kit is given to participants by study staff at these sites and mailed directly to the University of North Carolina at Chapel Hill (UNC). For other clinics (e.g., Gaudiani clinic), participants are sent emails about the study, receive saliva kits at home, and return them to UNC via post. For participants in MX, the saliva sample will be collected by research staff at a clinic or community event. Samples will be shipped to UNC. Standard DNA extraction and plating will be conducted at UNC, Queensland Berghofer Medical Research Institute (QIMR), and Karolinska Institutet (KI). Median DNA yield is 4 μg/ml saliva and excess saliva will be stored at − 20 °C. For DK, bloodspots linked to the DK national health registries will be genotyped by the DK Statens Serum Institut.

NZ has procedures in place for participants who reside in NZ and endorse Māori and/or Pasifika ethnicity consistent with current New Zealand ethics and best practice guidelines for health research. AU will adhere to national standards for data storage, access, and governance for participants from Aboriginal and Torres Strait Islander communities.

## Analyses

Using the most comprehensive state-of-the-science statistical analysis pipeline at the time of analysis, all analyses align into three phases reflecting Aims 2–4: i) interrogating heterogeneity and underlying biology of EDs, ii) risk prediction and identification of resilience and protective factors, and iii) determining where in the body EDs “live.” Careful consideration has gone into harmonizing multi-site cohorts and local ethics restrictions. US, MX, AU, and NZ (non-Māori/Pasifika), raw data will be stored on the UNC computing cluster “longleaf.” DK, SE, and NZ-Māori/Pasifika raw data cannot be shared and will be analyzed by respective sites into sharable summary statistics.

### Statistical Analyses of Heterogeneity and Underlying Biology of EDs (Aim 2)

#### Primary analyses (Aim 2a)

##### Genotyping, quality control (QC), and Imputation

EDs will be analyzed separately. Standard genotyping, QC, and imputation will be performed for US, MX, AU, and NZ. The genotyping array selected will be optimized for ancestrally diverse cohorts and downstream imputation accuracy. The Rapid Imputation for Consortias Pipeline (RICOPILI) [39] PGC GWAS analysis pipeline will be implemented and includes four key stages. Following best practices for multi-ancestry data [40], samples will be divided into ancestry specific cohorts, quality control and association testing performed, and finally meta-analyzed. Specifically, PCAiR GENESIS [41] with 1000 Genomes (1000G) reference panel [42] will be used to assign ancestry supergroups, PC-Relate will be used for relatedness checking [43], and admixture and ancestry assignments will be confirmed using ADMIXTURE [44]. Next, for each ancestry-specific cohort, genotype-QC will be performed, including estimating missingness, sample heterozygosity, sex concordance, Hardy–Weinberg equilibrium (HWE), minor allele frequency (MAF), batch effects, and cryptic relatedness between samples. The third stage will be phasing and imputation via Meta-imputation [45] using a high-quality, diverse reference panel, such as TOPMed [46] and expanded 1000G [47], to optimize imputation [48]. The fourth stage will be within-ancestry post-imputation QC, where cohorts will be filtered for imputation quality, MAF, and batch.

Genotyping, QC, and imputation will be performed in parallel at KI for all SE samples using parallel procedures, with minor variations as required. All DK samples will be genotyped by the DK Statens Serum Institut based on whole-genome-amplified DNA (in triplicate) and SNP array genotypes. QC and imputation will be performed in parallel at the Aarhus site. For NZ, Māori and Pasifika samples will be genotyped within NZ, with data QC and imputation also performed locally using parallel procedures, to honor sample and data sovereignty requirements. AU will follow similar procedures for Indigenous populations.

##### GWAS

GWAS will be performed by ancestry groups for each primary ED diagnosis, component behaviors, and dimensional traits captured through the EDGI2 battery. Binary and continuous GWAS will include correction for relevant covariates including sex, age, and genotype-derived principal components (PC) to correct for population stratification. The number of appropriate PCs will be determined using PCAiR.

For each ED, ancestry-specific EDGI2 GWAS will be meta-analyzed using MR-MEGA [49]. Where possible EDGI2 cohorts will be meta-analyzed with PGC-ED GWAS summary statistics, and any other publicly available ED GWAS summary statistics. In total, meta-analyses are expected to yield ~ 80,000 cases and ~ 730,000 controls (case-specific breakdown: AN≈47,000; BN≈16,000; BED≈10,000, ARFID≈6,500). By the time of analysis, additional cases may be contributed from independent international sites. Significant GWAS loci will be identified through standard per-GWAS Bonferroni correction and a conditional false discovery rate (FDR) approach across GWAS to identify shared loci [50].

##### Phenotypic and GSEM analyses

Case-case GWAS will explicitly compare ED cases (e.g., AN vs. BN; AN vs. BED etc.). Genomic structural equational modelling (GSEM) [51] will be applied to the GWAS summary statistics to identify common factors across EDs and between ED symptoms.

##### SNP-Heritability

We will estimate SNP-h^2^ with Genome-wide Complex Trait Analysis (GCTA) [52] based on individual genotypes to maximize accuracy. Covariate-adjusted LDSC [53] will be used to calculate partitioned SNP-h^2^ within homogenous and admixed populations using the best available population references for building LD scores (e.g., the latest high coverage 1000G whole genome sequencing [WGS] data) [47]. For discrete traits, GCTA and LDSC require an assumed lifetime population prevalence (K), and we will model a range of values to explore sensitivity.

##### Genetic Correlations

We will estimate *r*_*g*_ between ED traits and other psychiatric, metabolic, and anthropometric traits using LDSC and LAVA [54]. Since LDSC is limited to within-ancestry comparisons, we will apply POPCORN [55] and GCTA bivariate GREML [56] for trans-ancestry correlation analyses.

##### Polygenic Risk Score

PRS aggregate risk alleles across the genome, weighted for effect size, to provide an estimate of an individual’s risk for a disorder or trait. We will compare two methods, the classical *P*-value thresholding method and SBayesRC [57], and implement appropriate cross-population approaches, for example PRS-CSx [58]. Exclusion criteria for *P*-value thresholding include uncommon SNPs (MAF < 0.01), low quality variants (imputation INFO score < 0.7), indels, strand ambiguous SNPs, and SNPs within the highly variable extended MHC region (chr6:25–34 Mb). As standard, SNPs will be LD pruned and clumped. *P*-value thresholds are predefined (0.001, 0.01, 0.05, 0.1, 0.5). We will use SBayesRC to calculate PRS while modeling LD.

##### Rare CNV Analyses

To explore rare CNVs, we will use a clustering approach. CNVs will be called with validated complementary calling algorithms, iPattern [59], PennCNV [60], and QuantiSNP [61] in EnsembleCNV [62], which enables highly accurate CNV calling from SNP array data. Stringent QC include: exclusion of LogRDev Z > 2, BalleleDev Z > 2, or fragmented CNVs; samples with < 98% call rate, autosomal heterozygosity greater than five, unresolvable sex mismatch, unreliable calls (low-confidence score, < 10 probes, or only called by a single algorithm) will be excluded; and spurious calls will be filtered with QC CNV metrics, small CNVs, or CNVs where > 50% overlaps low confidence genomic regions, or hypervariable regions in white blood cells will also be excluded. Adjacent CNVs overlapping by > 75% will be annealed. Additional QC will be applied as necessary to DK bloodspots [63] and analysis may be limited to CNVs relevant to neuropsychiatric disorders (*N* = 53) [64–66]. Batch-wise Firth logistic regression (including covariates) will test CNV burden in cases and controls, followed by standardization of results with Stouffer’s signed Z-value meta-analysis. Rare CNV burden parameters include count, total and average length (kilobases [kb]), number of protein coding genes implicated, and total overlapped evolutionarily constrained bases [67]. Additionally, novel CNVs will be detected with breakpoint analyses via Firth’s logistic regression.

##### Bioinformatics analysis and integration

Bioinformatic analysis and integration will leverage existing data and code from previous PGC investigations and publicly available sources. As in previous ED GWAS (ANGI [2], EDGI [1]), initial gene enrichment analyses will be performed in the web browser-based application FUMA [68] as a starting point, due to its easily sharable graphical output, and then address seven major domains of bioinformatics integration. Basic SNP annotation includes evolutionary constraint scores for primates, mammals, and vertebrates, functional annotation of gene components, and enrichment for all major psychiatric GWAS results. Statistical fine mapping will be performed to identify putatively causal SNPs at each locus, using FINEMAP [69] and SuSie [70]. Common indel imputation will be performed with TOPMed and expanded 1000G imputation reference (as allowable) to enable investigation of putatively informative indels at locations such as gene regulatory regions. We will perform comprehensive gene annotation followed by enrichment analysis with stratified LDSC to address the need for contextual interpretation of GWAS loci. This will include annotating risk loci, in addition to the basic SNP annotations, whole exome sequencing (WES), and snRNA-seq for differential gene expression in psychiatric disorders, Mendelian inheritance patterns, and brain cell-type specific gene expression. Integration of GWAS data with functional genomics will be performed with available annotation databases of both bulk tissue and cell specific regulatory elements, chromatin regions, interaction regions, splicing and quantitative trait loci (QTL). For specific ED risk loci, we will emulate experimental evaluation of GWAS loci currently being undertaken for other psychiatric disorders, including the use of massively parallel reporter assays (MPRA), and dCas9 epigenome editing to experimentally validate functional consequences of risk loci.

##### Power

We have sufficient power (> 80%) for case–control GWAS. BN, BED, and ARFID, specifically, will have power to detect common variants (MAF > 5%) with modest effect sizes (ORs: ARFID > 1.27; BN > 1.22; BED > 1.17), and low-frequency variants (MAF > 1%) with relatively large effect sizes (odds ratios [ORs] > 1.68, 1.52, and 1.41, respectively). From ANGI and EDGI, AN GWAS already has sufficient power for detection of common variants, which EDGI2 will improve. This will be the first ED GWAS with sufficient power for case-case analyses. The largest case-case analysis will be able to detect common low-effect variants and low-frequency moderate-effect variants (OR > 1.18 and OR > 1.4, respectively), while the smallest case-case analysis will be able to detect common moderate effect variants, and low frequency large effect variants (OR > 1.35 and OR > 1.8). Analyses for heritability, *r*_*g*_, and PRS all have sufficient power.

### Patient stratification (Aim 2b)

Within-disorder heterogeneity, common in EDs, has both research and clinical implications [71–75]. Data driven taxonomy has improved disorder classifications previously [76]. We will mirror recent work using PRS to derive genetic clusters of schizophrenia phenotypic subtypes [77, 78] and apply advanced, unsupervised machine learning to group samples for clinically relevant outcomes, and compare these with ED PRS clusters. Standard steps include variable selection and normalization, Uniform Manifold Approximation and Projection (UMAP) [79] for dimensional reduction, and visualization and identify subgroups with HDBSCAN [80] density-based clustering. To ensure robustness and reliability, the clustering algorithm will be validated by applying the same procedures to both training and replication datasets (e.g., applying the same clustering algorithm across sites). Cluster similarity will be evaluated to assess the consistency and reproducibility of the clusters. Validated novel patient clusters will be compared to standard DSM-5 diagnoses with agreement matrices to identify whether clusters better explain clinical outcomes and heterogeneity, as well as demonstrate genetic diversity.

### Interrogating AN as a metabo-psychiatric trait hypothesis (Aim 2c)

#### Genetic correlations

We will replicate previously identified psychiatric, metabolic, and anthropometric correlations [3] using the EDGI2 cohort. We will follow the approach described in the primary analysis for *r*_*g*_. We will use the largest available GWAS data for heritable metabolic and anthropometric traits [81–90] at the time of the analysis and correct for multiple testing using FDR.

#### Pathway specific PRS

Pathway PRS is an extension of PRS that identifies how risk alleles aggregate in particular gene pathways and enables inference of mechanisms of genetic risk conferral. We will use PRSet [91] and established gene set repositories including GO, SynGO, KEGG, and REACTOME for cohort specific analysis, and validate findings against UK Biobank. We will perform single and multiple pathway associations within each ED and assess proportion of variance explained with a nested approach. All analyses will be corrected for ancestry PCs, age, and sex [92].

#### PheWAS

We will leverage existing UK Biobank data to test for relationships between clinical phenotypes and between ED-associated genetic variants and PRS. UK Biobank data include diet, medications, biochemical assays, exercise, substance use, anthropometry, and broad diagnostic histories. For each ED, PRS will be calculated for all participants in UK Biobank, and phenome-wide association studies (PheWAS) performed to test for associations between higher PRS and clinical and diagnostic data. QC of UK Biobank phenotypic data will include visualization of quantitative trait distribution, and tests for non-normality of distributions (heteroskedasticity). An FDR correction will be applied to correct for multiple testing. We will seek replication of significant findings in the All Of Us, Adolescent Brain Cognitive Development (ABCD), and PsychEMERGE cohorts [93].

#### Mendelian randomization

We will use bidirectional, two-sample MR to interrogate putative causal directions for metabolic and anthropometric traits with high *r*_*g*_ for AN. SNP instruments for MR testing of disorder pairs will be selected based on *p* < 5 × 10^–8^ and *p* < 5 × 10^–6^, balancing power and weak instrument bias [94]. Inverse variance weighting (IVW) [95] is the basic method, and additional standard test considerations include Steiger filtering [96] for pleiotropic SNPs, adjusting for overlap between GWAS with MRlap [97], and FDR. To further account for weak instrument, confounding, and pleiotropic bias, we apply MR-Egger [98], MR PARS [99], and debiased IVW [100] and MR-APSS [101].

#### Power

Genetic correlations between ED diagnoses and metabolic traits have > 99% power, and PRS with all variants have 100% and > 85% power, at small *P*-value thresholds. Where PRSet assumes 50 causal pathways, power is > 80%, and where 100 causal pathways are assumed, power is > 90%. For MR, under the conditions that metabolic GWAS cases > 50 k, GWAS_METAB_ beta > 0.05, AN R^2^ of SNP on exposure > 0.05 and OR > 1.1, power analyses indicate > 80% power to discover bi-directional causal relationships between AN and metabolic traits. Finally, PheWAS analyses [102] have sufficient power as selected biobanks meet the requirement of either > 200 cases or > 1,000 individuals with quantitative traits.

### Risk prediction (Aim 3)

A key outstanding question in complex trait genetics is how individuals with high genetic risk remain resilient (i.e., disease-free), while others with low genetic risk may develop disease. Here, we will compare high ED-PRS cases to low ED-PRS cases, and high ED-PRS cases to high ED-PRS controls to characterize ED risk and protective factors and parse the ‘gene x environment’ relationship [9]. For case-case comparison, we will meta-analyze case samples from EDGI, EDGI2, UK Biobank, and other biobanks. Low PRS cases will be investigated for environmental contribution to disorder development in the absence of high genetic risk. We will perform PheWAS with the following comparisons: top decile vs. bottom decile cases, top decile vs. all other cases, and low decile vs. all other cases. As pathway risk aggregation can differ from genome wide PRS, we will also compare pathway-PRS in high and low PRS cases to identify pathways with notable contribution to genetic risk or resilience. Similarly, we will then compare high PRS cases to high PRS controls to identify potentially protective environmental and genetic factors against disorder development despite the presence of overall genetic risk.

Finally, we will repeat clustering analyses as described in *‘Patient Stratification (Aim 2b)’*, using PRS scores as clustering variables in place of phenotype clustering [103]. ED GWAS (ANGI, EDGI, EDGI2, and ARFID Genes and Environment [ARFID-GEN]) are combined for maximum sample size, and PRS will be calculated for ED behaviors, psychiatric co-morbidities, and metabolic traits for each sample. PRS curve is nonlinear (disproportionally greater in those with the greatest PRS burden [104–106]) and will thus be categorized into quartiles as ordinal variables, from which Gower distance matrices will be derived and used as input for clustering. Our primary analysis will be case-based clustering but will additionally expand to case–control and control-only clustering (controls: ≥ 30 years old, no lifetime ED symptoms) for a more robust investigation. Clusters will be compared for diagnostic and symptomatic prevalence, and we will repeat PheWAS as described for PRS decile comparisons above.

### Determining where in the body EDs “live” (Aim 4)

To extrapolate GWAS-identified risk variants to clinically relevant biology, we will leverage growing functional datasets and dynamic tools. Our approach comprises three main stages: Gwas2cells (Aim 4a), disorder-relevant gene-tissue associations (Aim 4b), and ED-relevant environmental GREx modelling (Aim 4c). Since this is a rapidly developing area of genetics research, we will implement the highest quality available tools and datasets at the time of analysis. The first stage, Gwas2Cells, will identify ED-specific implicated brain cell and tissue types using stratified LDSC [107, 108]. The second phase will include transcriptomic imputation (TI) with genotype and expression data from post-mortem cohorts to predict GREx for the implicated tissue and cell types [109, 110]. TI approaches using raw data are robust to ancestry differences and eQTLs (expression QTLs) have higher conservation than matched SNPs [109, 111]. The third phase will incorporate environmental ED risk factors (BMI, sex [112] and stress) and identify ‘gene x environment’ interactions in a ‘dynamic TI’ model (dTI) [113]. Sex specific GREx will be calculated using TI models derived from sex-stratified data to calculate sex specific brain and body GREx. BMI GREx will be calculated with five different BMI categories, underweight (<18), low (18.5–19.9), average (20–24.9), borderline high (25.0–29.9), and high (≥ 30). Similarly, stress GREx will be modelled under low (0), average (2), and high life stress [10]. dTI can help us better understand how known risk factors including BMI, stress, and sex, individually and in tandem may drive expression of genetic risk factors.

### Translational summit (Aim 5)

The culmination of EDGI2 will be a translational summit, to directly address the gap between ED biological research and clinical intervention. We will facilitate interaction among key research leaders, community stakeholders, individuals with lived experience, experts with specialized knowledge in relevant cultural and clinical contexts, and funding bodies. The summit will be an intensive, full-day hybrid experience, incorporating presentations, round table discussions, and networking. Actionable outcomes of this summit will include a scientifically informed white paper summarizing clinical intervention and policy recommendations.

## Discussion

Over the past decade, ED genetics has made significant strides. The 2019 Watson et al. GWAS [3] opened the door for deeper inquiries regarding how genes related to both metabolic and anthropometric factors can impact risk for AN, independent of genes influencing BMI, and beyond the impact of genes related to other psychiatric disorders. Since then, we have documented a high twin-based heritability for ARFID [114] and conducted two large genomic studies of ARFID [115, 116], currently queued for genotyping. Moreover, EDGI samples (N ~ 18,000) from the US, AU, NZ, and DK are all queued for genotyping, which also contributes large numbers of samples from individuals from varied ancestries, improving the representativeness of ED GWAS.

EDGI2 extends our work both globally and scientifically. By setting high but achievable recruitment goals across affected populations, we will ensure that ED genetics includes genotypes from many populations to ensure that our results maximize the applicability of findings across different populations and enhance the scope of research participation to improve scientific understanding for all populations. By focusing on severe and/or longstanding EDs, we will identify individuals whose genomes may be enriched for causal alleles, while working toward clinical prediction models that incorporate genetic data that may enhance our ability to identify individuals at risk for poor outcomes early and to inform more personalized intervention. We will convert knowledge of genetic and environmental risk factors for EDs into biologically and clinically relevant and actionable insights. EDGI2 will generate new knowledge and yield an action plan for translating findings into biologically informed prevention and treatment interventions.

## Data Availability

US, MX, AU, NZ (NZ-non-Māori/non-Pasifika participant data): Deidentified data will be available from the National Data Archive (NDA; https://nda.nih.gov/) within 3 years of the end of the performance period and will be searchable using study number C5266. Biospecimens will be available from the NIMH Repository & Genomics Resource (NRGR; https://www.nimhgenetics.org/order-biosamples/how-to-order-biosamples) via Sampled. DNA samples will be searchable on NRGR’s website using the NRGR study number or diagnosis. Genomic data will be shared with controlled access in database of Genotypes and Phenotypes (dbGaP; https://www.ncbi.nlm.nih.gov/gap/) for health/medical/biomedical use, as allowed by the participant’s informed consents and the Institutional Certification. Genomic data will be findable and identifiable using a dbGaP study accession number. AU Aboriginal and Torres Strait Islander data: AU will adhere to national standards for data storage, access, and governance for participants from Aboriginal and Torres Strait Islander communities. NZ Māori and Pasifika data and samples: Genotype and phenotype data from Māori and Pasifika participants will be shared under controlled access after approval by the EDGI2-NZ data access committee. EDGI2-NZ Māori and Pasifika data will be harmonized with EDGI2 data from the US, MX, and others, thus allowing the data to be analyzed alongside data from other sites. Locations and access of the data and the EDGI2-NZ data access committee details will also be advised on the EDGI2 NZ website (edgi.nz), and in publications using the data. Additional information regarding samples can be obtained by contacting the NZ principal investigators (Jordan, Kennedy). SE: Genotype and phenotype data from EDGI2-Sweden will be shared under controlled access via Federated European Genome-phenome Archive (FEGA; https://fega.nbis.se/) Sweden. FEGA Sweden is hosted by the National Bioinformatics Infrastructure Sweden (NBIS) at SciLifeLab. The EDGI2 datasets will be findable through the European Genome-phenome Archive web portal. DNA samples will be available from the Swedish biobank with appropriate permissions and agreements. DK: Applications for and access to the Danish individual genotype data can be made by researchers through the following avenues. 1) Within GDPR compliant countries, individuals must have a nominal temporary employment at Aarhus University (4 hours per week) or at the Centre for Eating and Feeding Disorders based research (1 - 8 hours/week) Region Hovedstaden Psychiatry and use a university-owned computer to access individual-level Danish data. 2) Individual researchers must have a collaboration agreement and a data handling agreement with the National Center for Register-based research (Aarhus, Denmark). 3) Individual researchers must establish a collaboration agreement and a data handler agreement with the Mental Health Services of the Capital Region of Denmark (Copenhagen). N.B. Genomic summary results from this study can be shared through unrestricted access. Moreover, we will post R Markdown code to enable exact replication of our work.

## Abbreviations

1000G: 1000 Genomes
ABCD: Adolescent Brain Cognitive Development
AN: Anorexia nervosa
ANGI: Anorexia Nervosa Genetics Initiative
AN-RSI: Anorexia Nervosa Register-based Severity Index
AQ- 10: Autism Spectrum Quotient-10
ARFID: Avoidant/restrictive food intake disorder
ARFID-GEN: ARFID Genes and Environment
ASRS: Adult ADHD Self-Report Scale
AU: Australia
AUDIT: The Alcohol Use Disorders Identification Test
BED: Binge-eating disorder
BEDS- 7: Binge-Eating Disorder Screener (7 items)
BMI: Body mass index
BN: Bulimia nervosa
CDE: Common Data Elements
CET: Compulsive Exercise Test
CNV: Copy number variant
DK: Denmark
DIGS: Diagnostic Interview for Genetic Studies
DMS: Drive for Muscularity Scale
DNA: Deoxyribonucleic acid
DSM- 5: Diagnostic and Statistical Manual of Mental Disorders 5^th^ Edition
dTI: dynamic Transcriptomic imputation
DUDIT: The Drug Use Disorders Identification Test
ED: Eating disorder
ED100K.v1: Eating Disorders 100,000 Questionnaire Version 1
ED100K.v3: Eating Disorders 100,000 Questionnaire Version 3
ED100K.v4: Eating Disorders 100,000 Questionnaire Version 4
EDE-Q: Eating Disorders Examination-Questionnaire
EDGI: Eating Disorders Genetics Initiative
ED-QOL: Eating Disorders Quality of Life
EQ- 5D- 5L: EuroQol- 5 Dimension 5-level
eQTL: Expression quantitative trait locus
GAD- 7: Generalized Anxiety Disorder (7 item)
GCTA: Genome-wide Complex Trait Analysis
GLAD: Genetic Links to Anxiety and Depression study
GLP- 1: Glucagon-like peptide- 1
GREx: Genetically regulated gene expression
GSEM: Genomic structural equational modelling
GWAS: Genome-wide association study
HSI: Heaviness of Smoking Index
HWE: Hardy-Weinberg equilibrium
ICD: International Classification of Diseases
ISAS: Inventory of Statements About Self-Injury
IVW: Inverse variance weighting
Kb: Kilobases
KI: Karolinska Institutet
LD: Linkage disequilibrium
LDSC: LD score regression
LEC- 5: Life Events Checklist for DSM- 5
L-NIAS: Lifetime Version of the Nine Item ARFID Screener
L-PARDI-AR-Q: Lifetime Version of the Pica, ARFID, and Rumination Disorder Interview – ARFID – Questionnaire
MDDI: Muscle Dysmorphic Disorder Inventory
MAF: Minor allele frequency
MPRA: Massively parallel reporter assays
MPS: Multi-dimensional Perfectionism Scale
MR: Mendelian randomization
MX: Mexico
NIAS: Nine Item ARFID Screen
NSSI: Non-Suicidal Self-Injury
NZ: Aotearoa New Zealand
OCI-R: Obsessive Compulsive Inventory-Revised
OR: Odds ratio
PACE Questionnaire: Polygenic Risk of Anorexia Nervosa and its Clinical Expression Questionnaire
PARDI-AR-Q: Pica, ARFID, and Rumination Disorder ARFID Questionnaire
PCs: Principal components
PGC-ED: Eating Disorders Working Group of the Psychiatric Genomics Consortium
PheWAS: Phenome-wide association study
PHQ- 9: Patient Health Questonnaire- 9
PRS: Polygenic risk scores
PRSet: Pathway-based PRS analysis
QC: Quality control
QIMR: Queensland Berghofer Medical Research Institute
QOL: Quality of life
QTL: Quantitative trait loci
RCADS- 25: Revised Children’s Anxiety and Depression Scale- 25
r_g_: Genetic correlation
RICOPILI: Rapid Imputation for Consortias Pipeline
SE: Sweden
SITBI: Suicidal Ideation and Suicide Plan Sections of the Self-Injurious Thoughts and Behaviors Interview
SNP: Single nucleotide polymorphism
SNP-h^2^: SNP-heritability
snRNA-seq: Single-nucleus RNA sequencing
SSI: Statens Serum Institut
TI: Transcriptomic imputation
TOPMed: Trans-omics for Precision Medicine
UK: United Kingdom
UMAP: Uniform Manifold Approximation and Projection
UNC: University of North Carolina at Chapel Hill
US: United States of America
WGS: Whole genome sequencing
WHODAS: World Health Organization Disability Assessment Schedule

## Footnotes

1 BMI is included in this protocol as a widely used measure, we acknowledge its significant limitations and problematic use in the context of EDs. BMI was not designed as a health measure and fails to account for critical factors such as body composition, metabolic health, and other social and cultural factors. Importantly, its reliance as a diagnostic or evaluative tool can perpetuate weight stigma and obscure the complexities of EDs. The inclusion of BMI here is to align with established research norms while recognizing its inherent flaws.

## References

[1] Bulik CM, Thornton LM, Parker R, Kennedy H, Baker JH, MacDermod C, et al. The Eating Disorders Genetics Initiative (EDGI): study protocol. BMC Psychiatry. 2021;21(1):234. 33947359 10.1186/s12888-021-03212-3 PMC8097919

[2] Thornton L, Munn-Chernoff M, Baker J, Juréus A, Parker R, Henders A, et al. The Anorexia Nervosa Genetics Initiative (ANGI): Overview and methods. Contemp Clin Trials. 2018;74:61–9. 30287268 10.1016/j.cct.2018.09.015 PMC6338222

[3] Watson H, Yilmaz Z, Thornton L, Hübel C, Coleman J, Gaspar H, et al. Genome-wide association study identifies eight risk loci and implicates metabo-psychiatric origins for anorexia nervosa. Nat Genet. 2019;51:1207–14. 31308545 10.1038/s41588-019-0439-2 PMC6779477

[4] Termorshuizen J, Davies H, Lee S, Huckins L, Bulik C, Breen G, et al. Genome-wide association studies of binge-eating behaviour and anorexia nervosa yield insights into the unique and shared biology of eating disorder phenotypes. medRxiv. 2025. 10.1101/2025.01.31.25321397.

[5] Zickgraf HF, Ellis JM. Initial validation of the Nine Item Avoidant/Restrictive Food Intake disorder screen (NIAS): A measure of three restrictive eating patterns. Appetite. 2018;123:32–42. 29208483 10.1016/j.appet.2017.11.111

[6] Bryant-Waugh R, Stern CM, Dreier MJ, Micali N, Cooke LJ, Kuhnle MC, et al. Preliminary validation of the pica, ARFID and rumination disorder interview ARFID questionnaire (PARDI-AR-Q). J Eat Disord. 2022;10(1):179. 36419081 10.1186/s40337-022-00706-7 PMC9682666

[7] Birgegård A, Forsén Mantilla E, Dinkler L, Hedlund E, Savva A, Larsson H, et al. Validity of eating disorder diagnoses in the Swedish National Patient Register. J Psychiatr Res. 2022;150:227–30. 35398665 10.1016/j.jpsychires.2022.03.064

[8] Larsen J, Chatwin H, Clausen L, Bulik C, Thornton L, Micali N, et al. Construction of a register-based severity index for anorexia nervosa in Denmark: Association with overall and cause-specific mortality. medRxiv, https://www.medrxiv.org/content/10.1101/2025.02.14.25322233v1. 10.1192/j.eurpsy.2025.10059 PMC12344468 40670905

[9] Lindstedt K, Monell E, Birgegård A, Bulik CM, Termorshuizen JD, Clinton D (in press) Subjective experience of polygenic risk in anorexia nervosa: How do your genes feel? Psychiatric Genet. 10.1097/YPG.0000000000000395 40388529

[10] Herman BK, Deal LS, DiBenedetti DB, Nelson L, Fehnel SE, Brown TM. Development of the 7-Item Binge-Eating Disorder Screener (BEDS-7). Prim Care Companion CNS Disord. 2016;18(2). 10.1101/2023.12.27.573459. 10.4088/PCC.15m01896 PMC4956427 27486542

[11] Fairburn C, Beglin S. Eating Disorder Examination Questionnaire (6.0). In: Fairburn C, editor. Cognitive behavior therapy for eating disorders. New York: Guilford; 2008.

[12] Hildebrandt T, Langenbucher J, Schlundt DG. Muscularity concerns among men: development of attitudinal and perceptual measures. Body Image. 2004;1(2):169–81. 18089149 10.1016/j.bodyim.2004.01.001

[13] McCreary DR, Sasse DK. An exploration of the drive for muscularity in adolescent boys and girls. J Am Coll Health. 2000;48(6):297–304. 10863873 10.1080/07448480009596271

[14] U.S. Household Food Security Survey Module: Six-Item Short Form. In: US Department of Agriculture, 2012. https://www.ers.usda.gov/topics/food-nutrition-assistance/food-security-in-the-us/survey-tools

[15] Wootton BM, Diefenbach GJ, Bragdon LB, Steketee G, Frost RO, Tolin DF. A contemporary psychometric evaluation of the Obsessive Compulsive Inventory-Revised (OCI-R). Psychol Assess. 2015;27:874–82. 25664634 10.1037/pas0000075 PMC4530108

[16] Allison C, Auyeung B, Baron-Cohen S. Toward brief "Red Flags" for autism screening: The Short Autism Spectrum Quotient and the Short Quantitative Checklist for Autism in toddlers in 1,000 cases and 3,000 controls. J Am Acad Child Adolesc Psychiatry. 2012;51(2):202–12 e7. 10.1016/j.jaac.2011.11.003 22265366

[17] Kessler RC, Adler L, Ames M, Demler O, Faraone S, Hiripi E, et al. The World Health Organization Adult ADHD Self-Report Scale (ASRS): a short screening scale for use in the general population. Psychol Med. 2005;35(2):245–56. 15841682 10.1017/s0033291704002892

[18] Spitzer RL, Kroenke K, Williams JB, Löwe B. A brief measure for assessing generalized anxiety disorder: the GAD-7. Arch Int Med. 2006;166(10):1092–7. 16717171 10.1001/archinte.166.10.1092

[19] Kroencke K, Spitzer R, Williams J. The PHQ-9: validity of a brief depression severity measure. J Gen Int Med. 2001;16(9):606–13. 10.1046/j.1525-1497.2001.016009606.x PMC1495268 11556941

[20] Davies MR, Kalsi G, Armour C, Jones IR, McIntosh AM, Smith DJ, et al. The Genetic Links to Anxiety and Depression (GLAD) Study: Online recruitment into the largest recontactable study of depression and anxiety. Behav Res Ther. 2019;123: 103503. 31715324 10.1016/j.brat.2019.103503 PMC6891252

[21] Nock MK, Holmberg EB, Photos VI, Michel BD. Self-Injurious Thoughts and Behaviors Interview: development, reliability, and validity in an adolescent sample. Psychol Assess. 2007;19(3):309–17. 17845122 10.1037/1040-3590.19.3.309

[22] Nurnberger JI, Blehar MC, Kaufmann CA, York-Cooler C, Simpson SG, Harkavy-Friedman J, et al. Diagnostic Interview for Genetic Studies: rationale, unique features, and training. Arch Gen Psychiatry. 1994;51:849–59. 7944874 10.1001/archpsyc.1994.03950110009002

[23] Nicholson S, Jenkins R, Meltzer H. Adult psychiatric morbidity in England, 2007. The Information Centre for Health and Social Care. London; 2009. https://digital.nhs.uk/data-and-information/publications/statistical/adult-psychiatric-morbidity-survey/adult-psychiatric-morbidity-in-england-2007-results-of-a-household-survey

[24] Klonsky ED, Glenn CR. Assessing the functions of non-suicidal self-injury: Psychometric properties of the Inventory of Statements About Self-injury (ISAS). J Psychopathol Behav Assess. 2009;31(3):215–9. 29269992 10.1007/s10862-008-9107-z PMC5736316

[25] Cortés-Tomás M-T, Giménez-Costa J-A, Motos-Sellés P, Sancerni-Beitia M-D. Different versions of the Alcohol Use Disorders Identification Test (AUDIT) as screening instruments for underage binge drinking. Drug Alc Depend. 2016;158:52–9. 10.1016/j.drugalcdep.2015.10.033 26616473

[26] Heatherton TF, Kozlowski LT, Frecker RC, Rickert W, Robinson J. Measuring the heaviness of smoking: using self-reported time to the first cigarette of the day and number of cigarettes smoked per day. Br J Addict. 1989;84(7):791–800. 2758152 10.1111/j.1360-0443.1989.tb03059.x

[27] Berman AH, Bergman H, Palmstierna T, Schlyter F. Evaluation of the Drug Use Disorders Identification Test (DUDIT) in criminal justice and detoxification settings and in a Swedish population sample. Eur Addict Res. 2005;11(1):22–31. 15608468 10.1159/000081413

[28] Weathers F, Blake D, Schnurr P, Kaloupek D, Marx B, Keane T. The Life Events Checklist for DSM-5 (LEC-5). National Center for PTSD; 2013. https://www.ptsd.va.gov/professional/assessment/te-measures/life_events_checklist.asp

[29] Engel S. Health Related Quality of Life and Disordered Eating: Development and Validation of the Eating Disorders Quality of Life Instrument. [Unpublished Dissertation]. Fargo, ND: North Dakota State University; 2003.

[30] Engel S, Wittrock D, Crosby R, Wonderlich S, Mitchell J, Kolotkin R. Development and psychometric validation of an eating disorder-specific health-related quality of life instrument. Int J Eat Disord. 2006;39(1):62–71. 16345055 10.1002/eat.20200

[31] Taranis L, Touyz S, Meyer C. Disordered eating and exercise: development and preliminary validation of the compulsive exercise test (CET). Eur Eat Disord Rev. 2011;19(3):256–68. 21584918 10.1002/erv.1108

[32] Frost R, Marten P, Lahart C, Rosenblate R. The dimensions of perfectionism. Cog Ther Res. 1990;14(5):449–68.

[33] Narrow WE, Clarke DE, Kuramoto SJ, Kraemer HC, Kupfer DJ, Greiner L, et al. DSM-5 field trials in the United States and Canada, Part III: development and reliability testing of a cross-cutting symptom assessment for DSM-5. Am J Psychiatry. 2013;170(1):71–82. 23111499 10.1176/appi.ajp.2012.12071000

[34] Ustun TB, Chatterji S, Kostanjsek N, Rehm J, Kennedy C, Epping-Jordan J, et al. Developing the World Health Organization Disability Assessment Schedule 2.0. Bull World Health Organ. 2010;88(11):815–23. 10.2471/BLT.09.067231 PMC2971503 21076562

[35] National Institute of Mental Health. DSM-5 Self-Rated Level 1 Cross-Cutting Symptom Measure Youth Self Report. Department of Health and Human Services, editor. National Institute of Mental Health, National Data Archive. https://nda.nih.gov/data-structure/cde_dsm5crosspg01

[36] Chorpita BF, Yim L, Moffitt C, Umemoto LA, Francis SE. Assessment of symptoms of DSM-IV anxiety and depression in children: a revised child anxiety and depression scale. Behav Res Ther. 2000;38(8):835–55. 10937431 10.1016/s0005-7967(99)00130-8

[37] Herdman M, Gudex C, Lloyd A, Janssen M, Kind P, Parkin D, et al. Development and preliminary testing of the new five-level version of EQ-5D (EQ-5D-5L). Qual Life Res. 2011;20(10):1727–36. 21479777 10.1007/s11136-011-9903-x PMC3220807

[38] English MCW, Gignac GE, Visser TAW, Whitehouse AJO, Enns JT, Maybery MT. The Comprehensive Autistic Trait Inventory (CATI): development and validation of a new measure of autistic traits in the general population. Mol Autism. 2021;12(1):37. 34001225 10.1186/s13229-021-00445-7 PMC8130295

[39] Lam M, Awasthi S, Watson HJ, Goldstein J, Panagiotaropoulou G, Trubetskoy V, et al. RICOPILI: Rapid Imputation for COnsortias PIpeLIne. Bioinformatics. 2020;36(3):930–3. 31393554 10.1093/bioinformatics/btz633 PMC7868045

[40] Peterson RE, Kuchenbaecker K, Walters RK, Chen CY, Popejoy AB, Periyasamy S, et al. Genome-wide association studies in ancestrally diverse populations: Opportunities, methods, pitfalls, and Recommendations. Cell. 2019;179(3):589–603. 31607513 10.1016/j.cell.2019.08.051 PMC6939869

[41] Conomos MP, Miller MB, Thornton TA. Robust inference of population structure for ancestry prediction and correction of stratification in the presence of relatedness. Genet Epidemiol. 2015;39(4):276–93. 25810074 10.1002/gepi.21896 PMC4836868

[42] 1000 Genomes Project, Auton A, Brooks LD, Durbin RM, Garrison EP, Kang HM, et al. A global reference for human genetic variation. Nature. 2015;526(7571):68–74. 10.1038/nature15393 PMC4750478 26432245

[43] Conomos MP, Reiner AP, Weir BS, Thornton TA. Model-free estimation of recent genetic relatedness. Am J Hum Genet. 2016;98(1):127–48. 26748516 10.1016/j.ajhg.2015.11.022 PMC4716688

[44] Alexander DH, Novembre J, Lange K. Fast model-based estimation of ancestry in unrelated individuals. Genome Res. 2009;19(9):1655–64. 19648217 10.1101/gr.094052.109 PMC2752134

[45] Yu K, Das S, LeFaive J, Kwong A, Pleiness J, Forer L, et al. Meta-imputation: An efficient method to combine genotype data after imputation with multiple reference panels. Am J Hum Genet. 2022;109(6):1007–15. 35508176 10.1016/j.ajhg.2022.04.002 PMC9247833

[46] Taliun D, Harris DN, Kessler MD, Carlson J, Szpiech ZA, Torres R, et al. Sequencing of 53,831 diverse genomes from the NHLBI TOPMed Program. Nature. 2021;590(7845):290–9. 33568819 10.1038/s41586-021-03205-y PMC7875770

[47] Byrska-Bishop M, Evani US, Zhao X, Basile AO, Abel HJ, Regier AA, et al. High-coverage whole-genome sequencing of the expanded 1000 Genomes Project cohort including 602 trios. Cell. 2022;185(18):3426–40 e19. 10.1016/j.cell.2022.08.004 PMC9439720 36055201

[48] Xu J, Liu D, Hassan A, Genovese G, Cote AC, Fennessy B, et al. Evaluation of imputation performance of multiple reference panels in a Pakistani population. Hum Genet Genome Adv. 2025; 6(2). 10.1016/j.xhgg.2024.100395 PMC11759560 39696820

[49] Magi R, Horikoshi M, Sofer T, Mahajan A, Kitajima H, Franceschini N, et al. Trans-ethnic meta-regression of genome-wide association studies accounting for ancestry increases power for discovery and improves fine-mapping resolution. Hum Mol Genet. 2017;26(18):3639–50. 28911207 10.1093/hmg/ddx280 PMC5755684

[50] Smeland OB, Frei O, Shadrin A, O’Connell K, Fan CC, Bahrami S, et al. Discovery of shared genomic loci using the conditional false discovery rate approach. Hum Genet. 2020;139(1):85–94. 31520123 10.1007/s00439-019-02060-2

[51] Grotzinger AD, Rhemtulla M, de Vlaming R, Ritchie SJ, Mallard TT, Hill WD, et al. Genomic structural equation modelling provides insights into the multivariate genetic architecture of complex traits. Nat Hum Behav. 2019;3(5):513–25. 30962613 10.1038/s41562-019-0566-x PMC6520146

[52] Yang J, Lee SH, Goddard ME, Visscher PM. GCTA: a tool for genome-wide complex trait analysis. Am J Hum Genet. 2011;88(1):76–82. 21167468 10.1016/j.ajhg.2010.11.011 PMC3014363

[53] Luo Y, Li X, Wang X, Gazal S, Mercader JM, Me Research T, et al. Estimating heritability and its enrichment in tissue-specific gene sets in admixed populations. Hum Mol Genet. 2021;30(16):1521–34. 10.1093/hmg/ddab130 PMC8330913 33987664

[54] Werme J, van der Sluis S, Posthuma D, de Leeuw CA. An integrated framework for local genetic correlation analysis. Nat Genet. 2022;54(3):274–82. 35288712 10.1038/s41588-022-01017-y

[55] Brown BC, Asian Genetic Epidemiology Network Type 2 Diabetes C, Ye CJ, Price AL, Zaitlen N. Transethnic genetic-correlation estimates from summary statistics. Am J Hum Genet. 2016;99(1):76–88. 10.1016/j.ajhg.2016.05.001 PMC5005434 27321947

[56] Deary IJ, Yang J, Davies G, Harris SE, Tenesa A, Liewald D, et al. Genetic contributions to stability and change in intelligence from childhood to old age. Nature. 2012;482(7384):212–5. 22258510 10.1038/nature10781

[57] Zheng Z, Liu S, Sidorenko J, Wang Y, Lin T, Yengo L, et al. Leveraging functional genomic annotations and genome coverage to improve polygenic prediction of complex traits within and between ancestries. Nat Genet. 2024;56(5):767–77. 38689000 10.1038/s41588-024-01704-y PMC11096109

[58] Ruan Y, Lin YF, Feng YA, Chen CY, Lam M, Guo Z, et al. Improving polygenic prediction in ancestrally diverse populations. Nat Genet. 2022;54(5):573–80. 35513724 10.1038/s41588-022-01054-7 PMC9117455

[59] Pinto D, Darvishi K, Shi X, Rajan D, Rigler D, Fitzgerald T, et al. Comprehensive assessment of array-based platforms and calling algorithms for detection of copy number variants. Nat Biotechnol. 2011;29(6):512–20. 21552272 10.1038/nbt.1852 PMC3270583

[60] Wang K, Li M, Hadley D, Liu R, Glessner J, Grant S, et al. PennCNV: An integrated hidden Markov model designed for high-resolution copy number variation detection in whole-genome SNP genotyping data. Genome Res. 2007;17:1665–74. 17921354 10.1101/gr.6861907 PMC2045149

[61] Colella S, Yau C, Taylor J, Mirza G, Butler H, Clouston P, et al. QuantiSNP: an Objective Bayes Hidden-Markov Model to detect and accurately map copy number variation using SNP genotyping data. Nucleic Acids Res. 2007;35:2013–25. 17341461 10.1093/nar/gkm076 PMC1874617

[62] Zhang Z, Cheng H, Hong X, Di Narzo AF, Franzen O, Peng S, et al. EnsembleCNV: an ensemble machine learning algorithm to identify and genotype copy number variation using SNP array data. Nucleic Acids Res. 2019;47(7): e39. 30722045 10.1093/nar/gkz068 PMC6468244

[63] Calle Sanchez X, Helenius D, Bybjerg-Grauholm J, Pedersen C, Hougaard DM, Borglum AD, et al. Comparing copy number variations in a Danish case cohort of individuals with psychiatric disorders. JAMA Psychiat. 2022;79(1):59–69. 10.1001/jamapsychiatry.2021.3392 PMC8733851 34817560

[64] Coe BP, Witherspoon K, Rosenfeld JA, van Bon BW, Vulto-van Silfhout AT, Bosco P, et al. Refining analyses of copy number variation identifies specific genes associated with developmental delay. Nat Genet. 2014;46(10):1063–71. 25217958 10.1038/ng.3092 PMC4177294

[65] Marshall CR, Howrigan DP, Merico D, Thiruvahindrapuram B, Wu W, Greer DS, et al. Contribution of copy number variants to schizophrenia from a genome-wide study of 41,321 subjects. Nat Genet. 2017;49(1):27–35. 27869829 10.1038/ng.3725 PMC5737772

[66] Kendall KM, Rees E, Bracher-Smith M, Legge S, Riglin L, Zammit S, et al. Association of Rare Copy Number Variants With Risk of Depression. JAMA Psychiat. 2019;76(8):818–25. 10.1001/jamapsychiatry.2019.0566 PMC6583866 30994872

[67] Sullivan PF, Meadows JRS, Gazal S, Phan BN, Li X, Genereux DP, et al. Leveraging base-pair mammalian constraint to understand genetic variation and human disease. Science. 2023;380(6643):eabn2937. 10.1126/science.abn2937 PMC10259825 37104612

[68] Watanabe K, Taskesen E, Van Bochoven A, Posthuma D. Functional mapping and annotation of genetic associations with FUMA. Nat Commun. 2017;8(1):1–11. 29184056 10.1038/s41467-017-01261-5 PMC5705698

[69] Benner C, Spencer CC, Havulinna AS, Salomaa V, Ripatti S, Pirinen M. FINEMAP: efficient variable selection using summary data from genome-wide association studies. Bioinformatics. 2016;32(10):1493–501. 26773131 10.1093/bioinformatics/btw018 PMC4866522

[70] Wang G, Sarkar A, Carbonetto P, Stephens M. A simple new approach to variable selection in regression, with application to genetic fine mapping. J Royal Stat Soc Series B: Stat Method. 2020;82(5):1273–300. 10.1111/rssb.12388 PMC10201948 37220626

[71] Milos G, Spindler A, Schnyder U, Fairburn CG. Instability of eating disorder diagnoses: prospective study. Br J Psychiatry. 2005;187:573–8. 16319411 10.1192/bjp.187.6.573 PMC2710504

[72] Eddy KT, Dorer DJ, Franko DL, Tahilani K, Thompson-Brenner H, Herzog DB. Diagnostic crossover in anorexia nervosa and bulimia nervosa: implications for DSM-V. Am J Psychiatry. 2008;165(2):245–50. 18198267 10.1176/appi.ajp.2007.07060951 PMC3684068

[73] Castellini G, Lo Sauro C, Mannucc E, Ravaldi C, Rotella C, Faravelli C, et al. Diagnostic crossover and outcome predictors in eating disorders according to DSM-IV and DSM-V proposed criteria: a 6-year follow-up study. Psychosom Med. 2011;73:270–9. 21257978 10.1097/PSY.0b013e31820a1838

[74] Schaumberg K, Jangmo A, Thornton L, Birgegård A, Almqvist C, Norring C, et al. Patterns of diagnostic flux in eating disorders: a longitudinal population study in Sweden. Psychol Med. 2019;49:432–50. 10.1017/S0033291718001472 PMC6788452 29911514

[75] Tozzi F, Thornton L, Klump K, Bulik C, Fichter M, Halmi K, et al. Symptom fluctuation in eating disorders: correlates of diagnostic crossover. Am J Psychiatry. 2005;162:732–40. 15800146 10.1176/appi.ajp.162.4.732

[76] Neff RA, Wang M, Vatansever S, Guo L, Ming C, Wang Q, et al. Molecular subtyping of Alzheimer's disease using RNA sequencing data reveals novel mechanisms and targets. Sci Adv. 2021;7(2). 10.1126/sciadv.abb5398 PMC7787497 33523961

[77] Diaz-Papkovich A, Anderson-Trocme L, Gravel S. A review of UMAP in population genetics. J Hum Genet. 2021;66(1):85–91. 33057159 10.1038/s10038-020-00851-4 PMC7728596

[78] Lu Y, Kowalec K, Song J, Karlsson R, Harder A, Giusti-Rodríguez P, et al. Subtyping schizophrenia using psychiatric polygenic scores. medRxiv. 2023:2023.10.12.23296915.

[79] McInnes L, Healy J, Saul N, Gtroßberger L. UMAP: Uniform Manifold Approximation and Projection. J Open Source Software. 2018;3(29):861.

[80] Campello RJ, Moulavi D, Sander J, editors. Density-based clustering based on hierarchical density estimates. Advances in Knowledge Discovery and Data Mining: 17th Pacific-Asia Conference, PAKDD 2013, Gold Coast, Australia, April 14–17, 2013, Proceedings, Part II 17; 2013: Springer.

[81] Chen J, Spracklen CN, Marenne G, Varshney A, Corbin LJ, Luan J, et al. The trans-ancestral genomic architecture of glycemic traits. Nat Genet. 2021;53(6):840–60. 34059833 10.1038/s41588-021-00852-9 PMC7610958

[82] Lagou V, Magi R, Hottenga JJ, Grallert H, Perry JRB, Bouatia-Naji N, et al. Sex-dimorphic genetic effects and novel loci for fasting glucose and insulin variability. Nat Commun. 2021;12(1):24. 33402679 10.1038/s41467-020-19366-9 PMC7785747

[83] Khera AV, Chaffin M, Wade KH, Zahid S, Brancale J, Xia R, et al. Polygenic prediction of weight and obesity trajectories from birth to adulthood. Cell. 2019;177(3):587–96 e9. 10.1016/j.cell.2019.03.028 PMC6661115 31002795

[84] Xu J, Johnson JS, Signer R, Eating Disorders Working Group of the Psychiatric Genomics C, Birgegard A, Jordan J, et al. Exploring the clinical and genetic associations of adult weight trajectories using electronic health records in a racially diverse biobank: a phenome-wide and polygenic risk study. Lancet Digit Health. 2022;4(8):e604-e14. 10.1016/S2589-7500(22)00099-1 PMC9612590 35780037

[85] Christakoudi S, Evangelou E, Riboli E, Tsilidis KK. GWAS of allometric body-shape indices in UK Biobank identifies loci suggesting associations with morphogenesis, organogenesis, adrenal cell renewal and cancer. Sci Rep. 2021;11(1):10688. 34021172 10.1038/s41598-021-89176-6 PMC8139988

[86] May-Wilson S, Matoba N, Wade KH, Hottenga JJ, Concas MP, Mangino M, et al. Large-scale GWAS of food liking reveals genetic determinants and genetic correlations with distinct neurophysiological traits. Nat Commun. 2022;13(1):2743. 35585065 10.1038/s41467-022-30187-w PMC9117208

[87] Annese V. Genetics and epigenetics of IBD. Pharmacol Res. 2020;159: 104892. 32464322 10.1016/j.phrs.2020.104892

[88] Jung S, Kim Y, Park D, Lee Y, Park S, Baek J, et al. Case-case genome-wide association analysis identifying genetic loci with divergent effects on Crohn’s disease and ulcerative colitis. Hum Mol Genet. 2023;32(4):677–84. 36164742 10.1093/hmg/ddac241

[89] Gonzalez-Serna D, Ochoa E, Lopez-Isac E, Julia A, Degenhardt F, Ortego-Centeno N, et al. A cross-disease meta-GWAS identifies four new susceptibility loci shared between systemic sclerosis and Crohn’s disease. Sci Rep. 2020;10(1):1862. 32024964 10.1038/s41598-020-58741-w PMC7002703

[90] Cerqueira JXM, Saavalainen P, Kurppa K, Laurikka P, Huhtala H, Nykter M, et al. Independent and cumulative coeliac disease-susceptibility loci are associated with distinct disease phenotypes. J Hum Genet. 2021;66(6):613–23. 33446885 10.1038/s10038-020-00888-5 PMC8144013

[91] Choi SW, Garcia-Gonzalez J, Ruan Y, Wu HM, Porras C, Johnson J, et al. PRSet: Pathway-based polygenic risk score analyses and software. PLoS Genet. 2023;19(2): e1010624. 36749789 10.1371/journal.pgen.1010624 PMC9937466

[92] Zhou JY, Liu M, Park S. Interaction of environmental factors with the polygenic risk scores of thinness-related genes in preventing obesity risk in middle-aged adults: The KoGES. J Hum Nutr Diet. 2023;36:1451–67. 36632775 10.1111/jhn.13132

[93] Sealock JM, Lee YH, Moscati A, Venkatesh S, Voloudakis G, Straub P, et al. Use of the PsycheMERGE Network to investigate the association between depression polygenic scores and white blood cell count. JAMA Psychiat. 2021;78(12):1365–74. 10.1001/jamapsychiatry.2021.2959 PMC8529528 34668925

[94] Cheng Q, Yang Y, Shi X, Yeung KF, Yang C, Peng H, et al. MR-LDP: a two-sample Mendelian randomization for GWAS summary statistics accounting for linkage disequilibrium and horizontal pleiotropy. NAR Genom Bioinform. 2020;2(2):lqaa028. 10.1093/nargab/lqaa028 PMC7671398 33575584

[95] Lin Z, Deng Y, Pan W. Combining the strengths of inverse-variance weighting and Egger regression in Mendelian randomization using a mixture of regressions model. PLoS Genet. 2021;17(11): e1009922. 34793444 10.1371/journal.pgen.1009922 PMC8639093

[96] Hemani G, Tilling K, Davey SG. Orienting the causal relationship between imprecisely measured traits using GWAS summary data. PLoS Genet. 2017;13(11): e1007081. 29149188 10.1371/journal.pgen.1007081 PMC5711033

[97] Mounier N, Kutalik Z. Bias correction for inverse variance weighting Mendelian randomization. Genet Epidemiol. 2023;47(4):314–31. 37036286 10.1002/gepi.22522

[98] Burgess S, Thompson SG. Interpreting findings from Mendelian randomization using the MR-Egger method. Eur J Epidemiol. 2017;32(5):377–89. 28527048 10.1007/s10654-017-0255-x PMC5506233

[99] Zhao Q, Wang J, Hemani G, Bowden J, Small DS. Statistical inference in two-sample summary-data Mendelian randomization using robust adjusted profile score. 2018. arXiv preprint arXiv:1801.09652.

[100] Ye T, Shao J, Kang H. Debiased inverse-variance weighted estimator in two-sample summary-data Mendelian randomization. Ann Stat. 2021;49(4):2079–100. 10.1002/sim.10245 39453381

[101] Hu X, Cai M, Xiao J, Wan X, Wang Z, Zhao H, et al. Benchmarking Mendelian randomization methods for causal inference using genome-wide association study summary statistics. Am J Hum Genet. 2024;111(8):1717–35. 39059387 10.1016/j.ajhg.2024.06.016 PMC11339627

[102] Verma A, Bradford Y, Dudek S, Lucas AM, Verma SS, Pendergrass SA, et al. A simulation study investigating power estimates in phenome-wide association studies. BMC Bioinformatics. 2018;19(1):120. 29618318 10.1186/s12859-018-2135-0 PMC5885318

[103] Song J, Yao S, Kowalec K, Lu Y, Sariaslan A, Szatkiewicz JP, et al. The impact of educational attainment, intelligence and intellectual disability on schizophrenia: a Swedish population-based register and genetic study. Mol Psychiatry. 2022;27(5):2439–47. 35379910 10.1038/s41380-022-01500-2 PMC9135619

[104] Grove J, Ripke S, Als TD, Mattheisen M, Walters RK, Won H, et al. Identification of common genetic risk variants for autism spectrum disorder. Nat Genet. 2019;51(3):431–44. 30804558 10.1038/s41588-019-0344-8 PMC6454898

[105] Torkamani A, Wineinger NE, Topol EJ. The personal and clinical utility of polygenic risk scores. Nat Rev Genet. 2018;19(9):581–90. 29789686 10.1038/s41576-018-0018-x

[106] Wray NR, Lin T, Austin J, McGrath JJ, Hickie IB, Murray GK, et al. From basic science to clinical application of polygenic risk scores: A primer. JAMA Psychiat. 2021;78(1):101–9. 10.1001/jamapsychiatry.2020.3049 32997097

[107] Finucane HK, Reshef YA, Anttila V, Slowikowski K, Gusev A, Byrnes A, et al. Heritability enrichment of specifically expressed genes identifies disease-relevant tissues and cell types. Nat Genet. 2018;50(4):621–9. 29632380 10.1038/s41588-018-0081-4 PMC5896795

[108] Gazal S, Finucane HK, Furlotte NA, Loh PR, Palamara PF, Liu X, et al. Linkage disequilibrium-dependent architecture of human complex traits shows action of negative selection. Nat Genet. 2017;49(10):1421–7. 28892061 10.1038/ng.3954 PMC6133304

[109] Huckins LM, Dobbyn A, Ruderfer DM, Hoffman G, Wang W, Pardinas AF, et al. Gene expression imputation across multiple brain regions provides insights into schizophrenia risk. Nat Genet. 2019;51(4):659–74. 30911161 10.1038/s41588-019-0364-4 PMC7034316

[110] Gamazon ER, Wheeler HE, Shah KP, Mozaffari SV, Aquino-Michaels K, Carroll RJ, et al. A gene-based association method for mapping traits using reference transcriptome data. Nat Genet. 2015;47(9):1091–8. 26258848 10.1038/ng.3367 PMC4552594

[111] Highland HM, Wojcik GL, Graff M, Nishimura KK, Hodonsky CJ, Baldassari AR, et al. Predicted gene expression in ancestrally diverse populations leads to discovery of susceptibility loci for lifestyle and cardiometabolic traits. Am J Hum Genet. 2022;109(4):669–79. 35263625 10.1016/j.ajhg.2022.02.013 PMC9069067

[112] Oliva M, Munoz-Aguirre M, Kim-Hellmuth S, Wucher V, Gewirtz ADH, Cotter DJ, et al. The impact of sex on gene expression across human tissues. Science. 2020;369(6509). 10.1101/2023.12.27.573459. 10.1126/science.aba3066 PMC8136152 32913072

[113] Seah C, Signer R, Deans M, Bader H, Rusielewicz T, Hicks EM, et al. Common genetic variation impacts stress response in the brain. bioRxiv. 2023:2023.12.27.573459.

[114] Dinkler L, Lichtenstein P, Birgegard A, Bulik CM. Etiology of the broad avoidant restrictive food intake disorder phenotype in Swedish twins aged 6–12 years. JAMA Psychiat. 2023;80:260–9. 10.1001/jamapsychiatry.2022.4612 PMC9978946 36723946

[115] Bulik CM, Micali N, MacDermod CM, Qi B, Munn-Chernoff MA, Thornton LM, et al. ARFID Genes and Environment (ARFID-GEN): Study Protocol. BMC Psychiatry. 2023;23:863. 37990202 10.1186/s12888-023-05266-x PMC10664384

[116] Hog L, Fundin B, Palm E, Billger A, Bulik C, Abbaspour A, et al. ARFID InitiativE Sweden (ARIES): Study protocol. https://www.researchsquare.com/article/rs-4920763/v1. 10.1136/bmjopen-2024-095559 PMC12007039 40246566

